# Refined analysis of the Speech-to-Speech Synchronization task reveals subharmonic synchronization

**DOI:** 10.3389/fnins.2025.1611651

**Published:** 2025-07-02

**Authors:** Simon Bross, Andrea Hofmann, Kathleen Schneider, Isabell Wartenburger

**Affiliations:** Department of Linguistics, Cognitive Sciences, University of Potsdam, Potsdam, Germany

**Keywords:** Speech-to-Speech Synchronization, neural entrainment, Phase Locking Value (PLV), subharmonic synchronization, auditory-motor synchronization, speech processing, sensorimotor synchronization, hierarchical temporal processing

## Abstract

The Speech-to-Speech Synchronization task is a well-established behavioral approach to assess individual differences in auditory-motor synchronization. In this task, participants listen to a series of syllables that progressively increase in frequency, while simultaneously whispering the syllable /ta/ to synchronize with the rhythm of the incoming syllables. In our study, we replicated the bimodal distribution of high- and low-synchronizers in a sample of native German speakers. We present a refined analysis pipeline based on existing analysis scripts, address minor task-related issues and observations, and incorporate new analysis features such as the removal of silent gaps. Crucially, our analysis revealed that (sub-)harmonic interactions can emerge during various stages of synchronization and its assessment, obscured by the synchronization measurement. Subharmonic synchronizers were found to produce the /ta/-syllables to only every second or third incoming syllable which can result in deceptively high Phase Locking Values, thus challenging the conceptualization of low- and high-synchronizers. Our data analysis is available at OSF.

## 1 Introduction

Human speech processing relies on the intricate interplay between speech production and reception. Recent research has shown that neural oscillations involved in that interplay, particularly in the theta frequency range where the rhythmic organization of syllables occurs, are crucial for enabling the auditory cortex to track the speech envelope. These oscillations also facilitate auditory-motor synchronization, the coupling between the auditory and speech motor cortices (Assaneo et al., 2019). On this temporal mesoscale, the speech motor cortex interacts with auditory processes, where the syllable functions as a fundamental unit for encoding the coordinated articulatory movements necessary to create vocal tract constrictions (Poeppel and Assaneo, 2020).

Poeppel and Assaneo (2020) modeled the speech motor cortex as an oscillator with an inherently preferred rhythm, capable of generating rhythmic activity that entrains to external auditory input when the input frequency falls within a range centered around its preferred rhythm (~4.5 Hz). Neural entrainment of the auditory cortex is expected to facilitate speech segmentation, allowing the brain to parse continuous speech signals into meaningful units necessary for subsequent higher-order linguistic processes (Casas et al., 2021). This neural framework is not only compatible with harmonic synchronization (i.e., between frequencies in a 1:1 relationship) but also supports subharmonic synchronization, where neural activity entrains to input at subharmonic frequencies (1:m relationship, with m being an integer; Glass and Mackey, 1979). In sensorimotor synchronization tasks, subharmonic synchronization can be expected to emerge as a result of individual variability in adopting an alternative stable mode of (facilitated) synchronization.

The Speech-to-Speech Synchronization task (referred to as “task;” Assaneo et al., 2019; Lizcano-Cortés et al., 2022; Luo and Lu, 2023) is a frequently performed behavioral test to quantify the auditory-motor synchronization ability using an external auditory stimulus with either a steady (*Implicit Fixed* version) or an accelerating speech rhythm. In the *Explicit Accelerated* version that we employed, participants are presented with an external auditory stimulus containing an accelerating train of synthesized syllables increasing in frequency from 4.3 to 4.7 Hz. Participants are instructed to whisper the syllable /ta/ in line with the external syllable rate and usually perform two runs, preceded by a 10-second training phase. The task has been widely employed in different languages [e.g., English: (Assaneo et al., 2019; Orpella et al., 2022; Lubinus et al., 2023), German: (Assaneo et al., 2021; Rimmele et al., 2022; Oderbolz et al., 2024), Norwegian: (Sjuls et al., 2023), Mandarin: (Zhu et al., 2024), French: (Berthault et al., 2024), and Mexican Spanish: (Mares et al., 2023; Gómez Varela et al., 2024)].

These studies typically follow the published analysis approach (Lizcano-Cortés et al., 2022) that primarily focuses on computing the Phase Locking Value (PLV), measuring the synchronization between the external auditory stimulus and the speech output of participants, which reflects their auditory-motor coupling ability. Based on the PLV, studies reported bimodal distributions of high- and low-synchronizers. Despite the task being rather unnatural (whispering in line with a train of synthesized syllables without hearing their own audio-feedback), individuals who show high behavioral synchronization also demonstrate enhanced neural entrainment as evidenced by auditory cortical oscillations to the stimulus, and exhibit improved capabilities in word learning (Assaneo et al., 2019; Orpella et al., 2022). Therefore, the task and its investigated underlying mechanisms might have practical relevance for clinical and developmental populations (Assaneo et al., 2022; Ladányi et al., 2020; Eigsti and Pouw, 2024).

In our work, we aim to offer both theoretical and practical insights into the task, along with presenting additional avenues for analysis. Our refined analysis pipeline is based on the published analysis approach and further includes the implementation of (semi-)automated assessments and fine-tunings of the exclusion criteria, an acceleration-based segmented PLV, and a task-tailored method for estimating participants' syllable and articulation rates.

## 2 Method

### 2.1 Signal processing foundation

#### 2.1.1 Signal filtering

In (speech) signal processing, filters are leveraged to transform an input signal into an output signal by selectively attenuating or amplifying specific frequency components, thereby shaping the spectral characteristics. However, the inherent complexity of signals and the limitations of filters hinder a perfect filtering outcome, “so efforts to remove signal components either mean some of the undesired signal components remain, or that in removing the unwanted part of the signal other signal components are affected, or both” (Challis, 2021, p. 45).

A bandpass filter is designed to allow a certain frequency band pass—the passband—while it attenuates all other frequencies. For the task, this filter is applied to isolate the expected range of syllable rate frequencies from the participant and the speech envelope of the stimulus (4.3–4.7 Hz; the existing analysis framework used a 3.3–5.7 Hz passband for ±1 Hz tolerance). Such filtering introduces caveats as it preserves harmonic frequencies in the speech envelope at which no syllable production occurred.

**Figure 1:**
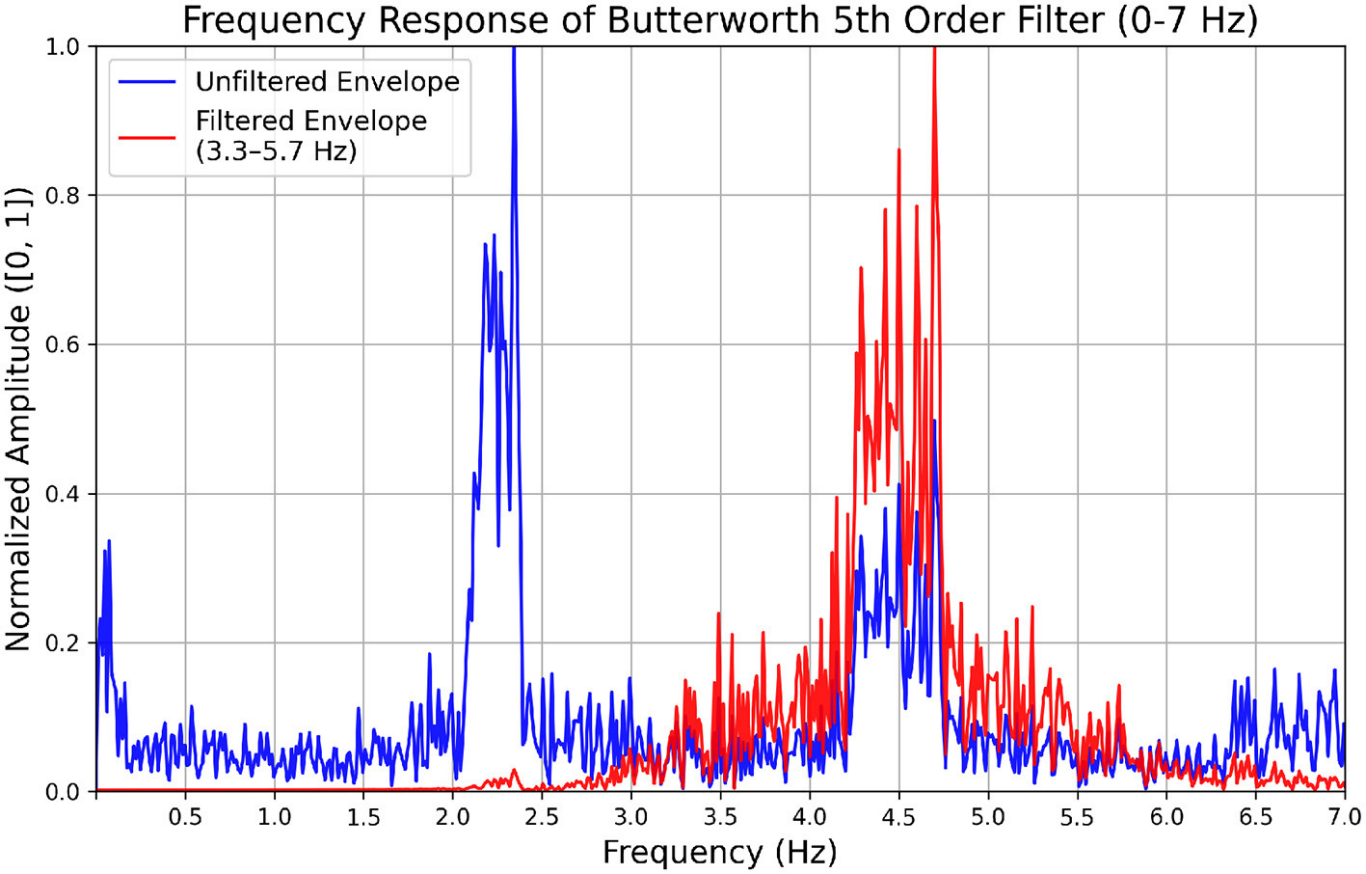
Effect of the bandpass filter on the modulation spectrum of the speech envelope of a subharmonic synchronizer (P2 run 1 in Supplementary Tables S1, S2) whispering at half the external syllable rate (i.e., consistently producing at the second subharmonic of the external syllable rate). The modulation spectrum was extracted from the speech envelope using the Fast Fourier Transform. The graph shows the modulation spectrum in the 0–7 Hz range before (blue) and after (red) applying a 5th order Butterworth bandpass filter (passband: 3.3–5.7 Hz) with a normalized amplitude between 0 and 1.

For instance, if a participant produces the /ta/ syllables to only every second syllable of the external auditory stimulus (e.g., at 2.25 instead of 4.5 Hz), it results in amplitude and phase modulations at 4.5 Hz in their speech envelope, which is the second harmonic of the participant's syllable production rate. Consequently, when applying a bandpass filter (3.3–5.7 Hz) to the speech envelope, the 2.25 Hz frequency is attenuated while the modulations at its second harmonic (4.5 Hz) that fall into the passband are preserved, leading to the impression that syllable production occurred at the second harmonic (see Figure 1). Disregarding these (sub-)harmonic interactions can result in misleading interpretations. We refer to participants showing this behavior as “subharmonic synchronizers”.

#### 2.1.2 The speech envelope

Speech is composed of thousands of frequencies whose energy levels fluctuate rapidly over time. The speech envelope (or amplitude envelope) of this complex signal refers to the slower amplitude fluctuations that mainly encode (supra-)segmental and prosodic features relevant for speech perception and rhythmic synchronization (Rosen, 1992; Goswami and Leong, 2013; Poeppel and Assaneo, 2020). Participants' speech envelopes are obtained by applying the Hilbert transform on their recordings, converting the speech signal into an analytic signal. The envelope is then extracted by computing the magnitude (absolute value) of this analytic signal, followed by the removal of the Direct Current offset, resampling, and bandpass filtering.

#### 2.1.3 Phase (Angles)

The local phase of a signal at a specific moment “describes the energy at that point, relative to the points before and after it. Unlike amplitude or loudness, which are absolute values, phase is an entirely relative property” (Leong, 2013, p. 72). Measured in angular units, the phase angle represents the fraction of a wave cycle completed at each sample of a signal (Alexander and Sadiku, 2008; Photinos, 2021). If unwrapped, the phase angle also displays how many cycles have been completed since the value does not reset after 2π but instead accumulates. In the bandpass-filtered speech envelope of the stimulus, one cycle (2π) corresponds to a single syllable, where cycle minima correlate with syllable onsets and maxima with syllable nuclei (Zhang et al., 2023).

The phase angle values and their timings are determined by the modulations at the syllable rate frequency. This information is used to assess the phase synchronization between the speech envelopes of the external auditory stimulus and the participant, for which the difference between their phase angle vectors is computed. In theory, if, for instance, at *t* both envelopes are at the same cycle count and exhibit a minimum, there is no phase difference between them.

#### 2.1.4 The Phase Locking Value

The PLV, introduced by Lachaux et al. (1999) as a measure of synchronization between neural signals, is used to quantify the synchronization between the speech envelopes of the auditory stimulus and the participant productions.

It is computed as follows:


$$\begin{array}{l} PLV=\frac{1}{T}|\overset{T}{\underset{t=1}{\sum }}e^{i\left(\theta_{1}\left(t\right)-\theta_{2}\left(t\right)\right)}| \end{array}$$

where *t* is the time index from 1 to *T* (total number of samples), θ_1_(*t*) and θ_2_(*t*) the phase angle vectors of two signals (here: filtered speech envelopes), and $e^{i\left(\theta_{1}\left(t\right)-\theta_{2}\left(t\right)\right)}$ the phase difference between them, mapped onto the unit circle in the complex plane. The PLV ranges from 0 to 1; a value of 1 indicates perfect phase locking (the phase difference between two signals remains perfectly consistent across *T*) and a PLV of 0 indicates no phase locking.

In essence, the PLV represents the phase difference consistency over time (cf. Supplementary Figure S6), meaning that despite a temporal shift between two signals with the same frequency, they still exhibit a high PLV if the phase difference between them remains stable (i.e., are phase-locked). This allows participants to produce their syllables with a certain positive or negative response lag: If participants produce the syllables steadily and with the same frequency compared to the external auditory stimulus, the phase relationship between them remains constant. For instance, if both external auditory stimulus and participant would produce at exactly 4.3 Hz and the participant demonstrates a perfectly consistent response lag of x ms for each syllable, the PLV will nonetheless amount to 1. The PLV plunges once this response lag varies over time or if there is a mismatch between the produced and external syllable rate frequency resulting in phase difference fluctuations.

However, there is an edge case for subharmonic synchronizers. As outlined in Section 2.1.1, the filter attenuates the 2.25 Hz frequency but preserves its second harmonic at 4.5 Hz. In the filtered speech envelope of the participant, two oscillations thus correspond to just one produced syllable. This is misleading because the PLV is computed between 4.5 (second harmonic) and 4.5 Hz instead of between 2.25 (produced syllable rate frequency) and 4.5 Hz, leading to high PLVs because both frequencies now seemingly coincide (cf. Supplementary Figure S1).

### 2.2 Participants

Sixty adults participated [48 females, 12 males; age range: 18–49 years, mean age: 24.78 years, standard deviation (SD): 6.07]. Inclusion criteria were: German native speakers, no history of speech or language disorders, hearing impairments, neurological, or psychological conditions. All participants completed two runs of the *Explicit Accelerated* version of the task with a stimulus length of 80 s, preceded by a 10 s test phase. The stimulus was taken from Assaneo (2022).

### 2.3 (Refined) exclusion criteria

The following gathers the exclusion criteria for the task that were established in Assaneo et al. (2019), the protocol (Lizcano-Cortés et al., 2022), and the Python script by Mares (2022). We refined specific criteria because they were originally designed for data collected via the internet, whereas our participants were recorded in a soundproof booth in a highly controlled laboratory setting.

**Figure 2:**
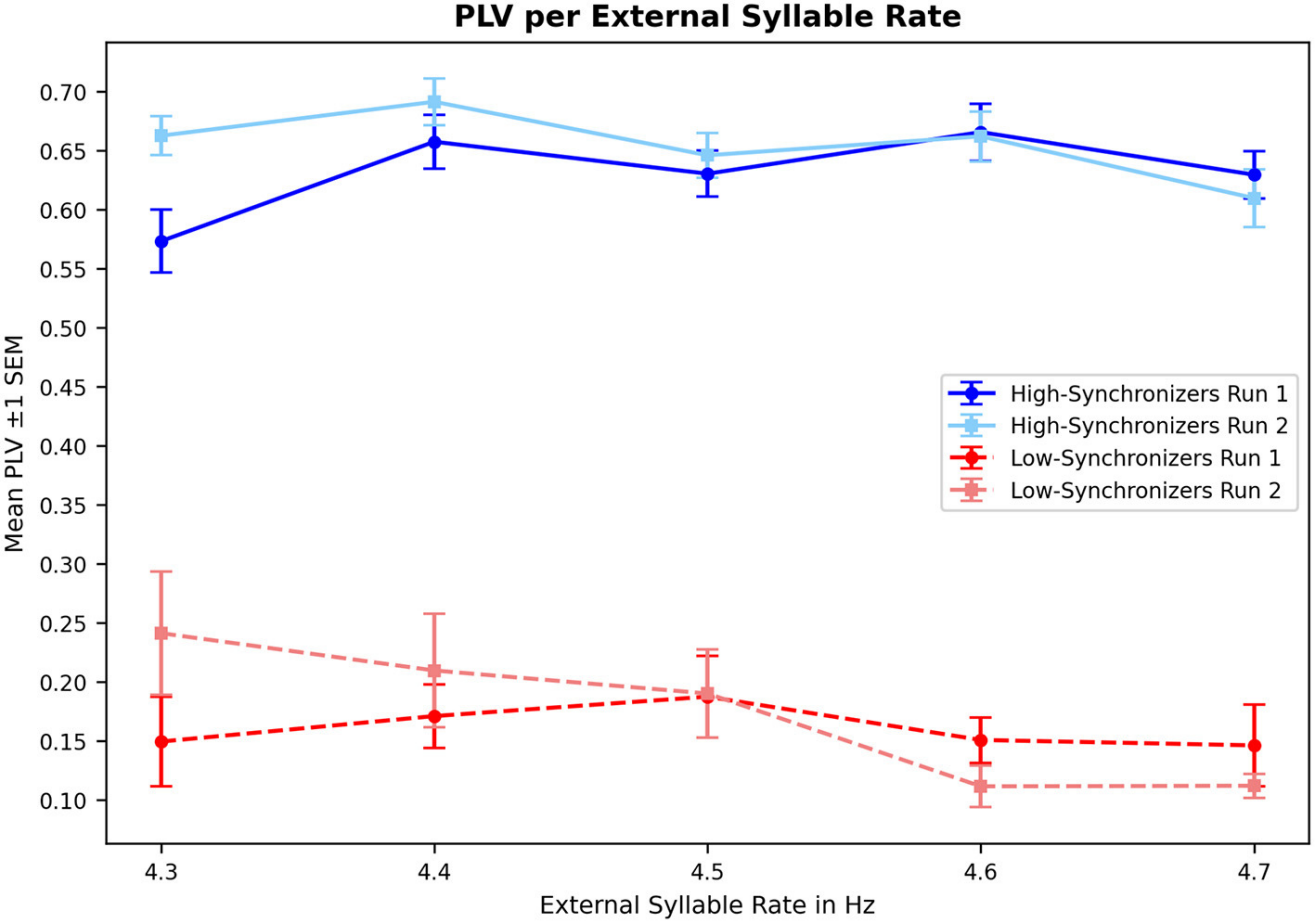
Mean PLVs and standard error of the mean (SEM) per segment (5 segments, cf. Section 2.4.4) for high- and low-synchronizers in run 1 and 2. Averaged across runs, high-synchronizers show peak synchronization at 4.4 Hz (segment 2), while low-synchronizers do so at 4.3 Hz (segment 1).

1. **PLV thresholds**: Participants exhibiting PLVs above 0.9 or below 0.1 in any of the two task runs should be excluded because these values may indicate potential artifacts or lack of participant engagement. We adhere to this criterion.
2. **Consistency between runs**: A linear regression model (Mares, 2022) predicts the expected PLV outcome of the second run based on the first. Participants should be excluded if the PLV of the second run deviates significantly from the predicted value (beyond 1.96 standard deviations). Our approach aims to retain participants whose PLVs from both runs fall into the same category (i.e., either high- or low-synchronizer in both runs), excluding only those who do not meet this criterion (cf. Section 2.4.6). Another viable strategy involves analyzing only the data from the second run, since we observed that participants frequently encounter initial difficulties in the first run where they acclimate to the task, hence treating the first run as a test run (cf. Figure 2).
3. **Environmental noise**: Participants should be excluded if environmental noise obscures the reconstruction of the speech envelope required for PLV computation. Recordings must be listened to once in order to identify environmental noise.
4. **Silent gaps**: Participants should be excluded if they show silent gaps between syllables exceeding three seconds. Our approach automatically detects and removes them instead (cf. Section 2.4.1). It may be beneficial to further remove gaps ≤ 3 s, as removing only gaps ≥ 3 s overlooks multiple shorter gaps that together exceed this threshold and are mainly due to physiological requirements (breathing). Silent gaps caused by breathing can be prevented by demonstrating participants how to produce syllables while inhaling.
5. **Speech mode**: Participants who speak loudly instead of whispering for longer than 3 or 4 s should be removed. While we did not observe this behavior in our data, our script provides a functionality to manually remove voiced segments (or other segments, e.g., non-detected silent gaps) from the participant data. The identification of non-whispered segments could also be automated using F0 detection.
6. **Articulation rate**: Participants should be excluded if they exhibit a “spoken rate” ≤ 2 Hz (i.e., the participant produces two /ta/s or fewer per second, including silent gaps). Our analysis provides an estimation for the articulation rate instead (excluding silent gaps; cf. Section 2.4.5). Nevertheless, we refrained from applying this criterion because any potential disengagement could be monitored in our controlled setting, ensuring that articulation rates ≤ 2 Hz are most likely indicative of low synchronization capabilities.
7. **Leaking stimulus**: If the external auditory stimulus leaks into the participant recording, remaining audible even after applying a bandstop filter, the participant should be excluded.

### 2.4 Refined data analysis pipeline

#### 2.4.1 Detection and removal of silent gaps

We defined silent gaps as time spans longer than 3 s during which no syllable onset /t/ is detected. Out-of-the-box implementations for automatic syllable or pause detection proved unreliable as they depend on F0 detection—absent in whispered speech—or on amplitude-based features unsuitable for the generally low but highly variable intensity in whispered speech. Instead, we used the spectral flux onset strength envelope (capturing dynamic changes in energy across frequencies) from the librosa Python package (McFee et al., 2025) to identify the recurring transient /t/ onsets in the participant recordings. Silent gaps are carefully removed (cf. Section 2.4.3).

#### 2.4.2 Stimulus leakage removal

Despite careful instructions and controlled settings, the stimulus can leak into the participant recording and distort results. We observed that external stimulus leakages introduce an F0 of 200 Hz into the recordings, indicating a potential for automatic leakage detection. Since the spectral content of the produced /ta/ syllables typically exceeds 3 kHz and the stimulus is ideally bandpass filtered between [0, 3 kHz], leakages can be removed using a bandstop filter with a [0, 3 kHz] stopband (Lizcano-Cortés et al., 2022). We used a Chebyshev 2 filter of 6th order and 40 dB attenuation in the stopband for this task. Post-filtered recordings must be carefully checked since participant productions might be filtered out if produced below 3 kHz (we provide a sanity-script to visually validate the reconstruction of the post-filtered recording/speech envelope, cf. Supplementary Figure S2).

#### 2.4.3 Corrected moving window PLV

We encountered a small error in the PLV computation of Mares' (2022) Python analysis script (Script name: funcs.py, function: PLVevol). The time and plv variables in the script are initialized based on a wrong number of expected windows, leading to an excess window that is not assigned a value (initialized with and remains at 0) but is considered when taking the mean across all moving windows. We noticed this because we computed the PLV between the stimulus and itself for testing purposes, which yielded a value of 0.975 instead of 1. Furthermore, the parameters for the window and overlap duration could not be set to values other than the defaults—changing them throws an IndexError. We corrected both issues.

Our data analysis uses the same window parameters as set in Mares' script (window size of 5 s with a 3 s overlap). Silent gaps equal to or longer than three seconds are removed prior to computation. It is pivotal to remove the respective samples from both the stimulus and participant only once the unwrapped phase angle vectors are extracted.

While the moving overlapping window approach captures short-term fluctuations in synchronization, it presents certain drawbacks. Firstly, it is not able to capture long-term synchronization and how well participants respond to the syllable rate changes in the external auditory stimulus. Secondly, overlapping windows results in double computations of segments that are not averaged out, possibly in- or deflating the PLV. To mitigate these drawbacks, an acceleration-based segmented PLV is presented.

#### 2.4.4 Acceleration-based segmented PLV

We observed that the 80 s version of the accelerating stimulus—also the available 60 s version—does not accelerate every 10 seconds, as described in Lizcano-Cortés et al. (2022), but after around 13–14 seconds, see below. According to the MBROLA script with which the stimulus was created, the stimulus is composed of the following five segments:

1. Segment (0–~13.95 s): 4.3 Hz (120 phonemes a ca. 116.28 ms)
2. Segm. (~13.95–~27.59 s): 4.4 Hz (120 phonemes a ca. 113.64 ms)
3. Segm. (~27.59–~40.92 s): 4.5 Hz (120 phonemes a ca. 111.11 ms)
4. Segm. (~40.92–~53.97 s): 4.6 Hz (120 phonemes a ca. 108.70 ms)
5. Segm. (~53.97–~80.14 s): 4.7 Hz (246 phonemes a ca. 106.38 ms)

We used these segmentations (i.e., 5 segments, cf. Figure 2) to compute the acceleration-based segmented PLV, with silent gaps ≥3 s removed from the segments, and segments in which less than 50% of the original data remains after removal are assigned a NaN. The PLVs for the segments are computed without overlapping windows to assess the participants' adaption and synchronization consistency to the external syllable rates.

While this segment-based approach directly correlates the PLV with external syllable rates, it may miss finer temporal details within segments due to the lack of overlapping windows. It can therefore be advisable to use both the moving window and segmented PLV in a complementary approach.

#### 2.4.5 Syllable and articulation rate estimation

The syllable rate is often estimated using the peak frequency of the speech envelope modulation spectrum. However, findings question its reliability: “[...] [T]he peak frequency of the narrowband modulation spectrum is not strongly influenced by the rate of syllables, but instead may originate from the biophysical properties of the human articulator” (Zhang et al., 2023, p. 8). They further found that “local features in the speech envelope, instead of the modulation spectrum, are a more reliable acoustic correlate of syllable” and that the peak frequency is only reliable “[...] when the analysis is pooled over minutes of speech recordings” (Zhang et al., 2023, p. 1). Our data confirmed that this approach often produced inaccurate syllable rate estimates per segment for the participant data, hence we desisted from using it. For other approaches, consult, for instance, Jadoul et al. (2016), Coupé et al. (2019), and MacIntyre et al. (2022).

Since the synchronization measurement of this task is not based on the syllable rate in terms of syllables per second but on the modulations in the frequency domain determined by the speech rhythm, we took advantage of the expected inherent rhythmicity and repurposed the PLV as a similarity metric between the participant's speech envelope and synthesized signals that represent hypothetical syllable rate frequencies. As a preliminary step, we filtered the participant's speech envelopes between 1–5 Hz to capture produced syllable rate frequencies within that range and synthesized signals from 1–5 Hz in 0.05 Hz increments. Using the PLV, we compared each segment (*n* = 5) of the participant speech envelope (silent gaps ≥1.5 s removed to approximate phonation time) with the synthesized signals and determined the best match, that is, the frequency that showed the highest PLV, indicating the most prominent rhythmic alignment.

Based on the preliminary results, estimations were refined by filtering the speech envelope in narrower bands (either between 1–3.3 Hz or 2.7–5 Hz) to improve accuracy. The resulting five values for the estimated syllable rates thus represent the dominant rhythmic frequency associated with syllable production and the overall articulation rate was calculated by weighting these values by the corresponding phonation time, thus estimating the number of syllables produced per second during active vocalization. While this approach can slightly overestimate the results for synchronizers with highly variable inter-onset-intervals, it remains a valid metric because it captures dominant phase-aligned periodicities by using smaller overlapping windows rather than requiring strict isochrony.

#### 2.4.6 Participant classification

To classify a participant run as either high or low, we extracted the parameters from the original analysis' Gaussian Mixture Model (Lizcano-Cortés et al., 2022; Mares, 2022) for determining the PLV classification thresholds, enhancing data comparability across studies.

Runs are classified as follows:


$$\begin{array}{ll} \text{Unreliable:} & \;\text{PLV}<0.1\text{ or PLV}>0.9\\ \text{High:} & \;0.4113<\text{PLV}\leq 0.9\\ \text{Low:} & \;0.1\leq \text{PLV}<0.4014\\ \text{Border case:} & \;0.4014\leq \text{PLV}\leq 0.4113 \end{array}$$

Border cases emerge from the intersection of high- and low-sychronizers in the model, making classification ambiguous. Border cases should be checked manually as described in Section 3.1 and, if possible, reclassified.

## 3 Results

### 3.1 Participant exclusion and classification

Four participants were excluded based on our refined PLV inconsistency criterion (cf. Section 2.3), all other (refined) exclusion criteria were not applied. If we would have applied the unmodified exclusion criteria together with the erroneous PLV computation, 19 out of the 60 participants would have been excluded (6 due to an articulation rate ≤ 2 Hz, 8 due to silent gaps ≥ 3 s, 7 due to incongruent PLVs across runs; 2 were affected by 2 criteria at once).

In summary, 56 participants (44 females) from the sample remained after applying the modified exclusion criteria (mean age: 24.5 years, SD: 5.95).

The PLVs are bimodally distributed with 12 low- and 44 high-synchronizers (see Supplementary Figure S3). One participant was classified as a border case in run 1 (PLV: 0.409) and as a low-synchronizer in run 2 (PLV: 0.222). Since leaning toward the threshold for low-synchronizers in the first run, we manually re-classified them as a low-synchronizer. As stated, we only include participants that are assigned the same class in both runs. For comparison, Supplementary Figure S4 shows the bimodal distribution for the whole sample (*N* = 60).

### 3.2 Moving window and segmented PLV

Mean moving window PLVs (silent gaps removed) across run 1 and 2 range from 0.164 to 0.857 (mean: 0.585, SD: 0.194). Despite the 10 s training phase prior to the first run, the segmented PLV corroborates participants' starting difficulties in the initial segment of the first run, especially in the high-synchronizers (cf. Figure 2; for a comparable Figure showing only the subharmonic synchronizers see Supplementary Figure S5).

Descriptively, high-synchronizers seem to better maintain synchronization across all frequencies—even for the longer 4.7 Hz segment—while low-synchronizers show decreasing PLVs for faster rates (cf. also Supplementary Figure S6). Comparing the mean moving window PLV (high-synchronizers: 0.678, low-synchronizers: 0.259) and the mean of the weighted average segmented PLV (*n* = 5 segments, weights based on remaining signal length per segment after silent gap removal; high-synchronizers: 0.638, low-synchronizers: 0.161) across both runs, the moving window approach yields statistically significantly higher values in both high- and low-synchronizers (highs: t = 18.018, *p* < 0.01; lows: t = 12.87, *p* < 0.01), likely due to its smoothing effect and not being strictly correlated with the segments of the external syllable rates.

### 3.3 Syllable and articulation rates

Articulation rates for high-synchronizers ranged from 2.24 to 4.56 Hz (mean = 4.29 Hz) in the first run and from 2.25 to 4.6 Hz (mean = 4.42 Hz) in the second run. For low-synchronizers, the articulation rates varied from 1.34 to 4.29 Hz with a mean of 2.58 Hz in the first run, and from 1.24 to 4.24 Hz with a mean of 2.72 Hz in the second run. Low-synchronizers' articulation rates thus deviate more significantly from the articulation rate of the external auditory stimulus (~4.5 Hz), resulting in their low PLVs. Unexpectedly, high-synchronizers exhibit a wide range of articulation rates due to presence of subharmonic synchronizers (i.e., synchronizing to only every second syllable, yielding articulation rates around 2.25 Hz). However, with the exception of one participant (articulation rate: 4.6 Hz), they never exceed the articulation rate of the external auditory stimulus. This suggests that the external rates serve as an upper synchronization bound. The syllable rate estimation per segment allowed us to identify 11 subharmonic synchronizers with variable behavior across runs (cf. Supplementary Tables S1, S2). Subharmonic synchronizers are also more descriptively discussed in Supplementary Section 3.

## 4 Discussion

This article provides practical and theoretical considerations and refinements for the analysis of the Speech-to-Speech Synchronization task performed under well-controlled laboratory conditions. Our analysis comprises the correction of the moving window PLV computation, the presentation of a segmented PLV, and functionalities to cope with stimulus leakages. Furthermore, we incorporate the automatic detection and removal of silent gaps, the possibility to semi-automatically remove non-whispered or other undesired segments from the participant signal, and adaptions to the exclusion criteria which previously would have led to considerable participant exclusion in our sample. Most importantly, the refinements allowed us to carve out previously concealed synchronization patterns beyond the binary classification into high- and low-synchronizers, and to retain 56 participants (out of 60) instead of 41 when following the original analysis. This is most relevant for clinical or developmental samples which are typically smaller in size—and therefore rely strongly on participant inclusion—but also more likely to exhibit difficulties adhering to the experiment instructions.

The exclusive reliance on the PLV in the original analysis can be misleading, as it may obscure individual synchronization dynamics and particularly (sub-)harmonic interactions occurring at various levels, such as the filter, syllable production, or neural level. In neuronal systems, (sub-)harmonic synchronization enables oscillators to process information and to communicate between different brain regions (Ermentrout, 1981; Pikovsky et al., 2001; Canolty and Knight, 2010). Subharmonic syllable synchronization can be either explained by subharmonic entrainment of the auditory cortex to the external auditory stimulus or by subharmonic entrainment of the speech motor cortex to the input from the auditory cortex. Although some participants transitioned from 1:2 synchronization in the first run to 1:1 synchronization in the second run, it remains unclear, without further examination, whether subharmonic synchronizers exhibit what could be termed impaired auditory entrainment.

Assuming the hypothesis that the speech motor cortex acts as an oscillator (Assaneo and Poeppel, 2018), one could expect it to demonstrate subharmonic synchronization as well. Cross-frequency coupling in subharmonic synchronization can be described and assessed by the n:m PLV (Vasudeva et al., 2022, cf. Supplementary Table S2). In the task context, this is evident in participants synchronizing to only every m-th syllable because the speech motor cortex might n:m phase-lock to the input from the auditory cortex. The existence of subharmonic synchronizers therefore provides support for modeling the speech motor cortex as an oscillator.

Leveraging the PLV in this task also questions the simplistic dichotomous classification into low- and high-synchronizers because not only can the mere reliance on this metric hide individual synchronization dynamics, but also both harmonic (1:1) and subharmonic (1:m) syllable synchronization can unnoticeably lead to high PLVs. Is “high synchronization” defined by the ability to consistently produce each syllable in sync (1:1), or rather by the general capacity of the speech motor cortex to entrain to the input from the auditory cortex, including at harmonic (1:1) and subharmonic (1:m) ratios? To further test subharmonic synchronization and the hypothesis that the speech motor cortex acts as an oscillator, future research could perform the task with the instruction to 1:m synchronize to the external auditory stimulus, assessing synchronization with the n:m PLV. Since the subharmonic rates correspond roughly to suprasegmental features in speech, their revelation in this study supports the view that the sole focus on syllables and the theta frequency range in auditory-motor synchronization (tasks) might be too narrow, neglecting both higher-order linguistic structures and hierarchical temporal processing.

## Data Availability

The data analyzed in this study is subject to the following licenses/restrictions: data will be released with the other study they were collected for. They are not relevant for this article. The code, relevant for this article, will be made available on OSF. Requests to access these datasets should be directed to isabell.wartenburger@uni-potsdam.de.
