# Supplementary material for: Refined analysis of the Speech-to-Speech Synchronization task reveals subharmonic synchronization
Source: Front Neurosci. 2025 Jul 2;19:1611651. doi: 10.3389/fnins.2025.1611651 (PMC12263569; doi:10.3389/fnins.2025.1611651)
Supplement: Supplementary file 1 [file Data_Sheet_1.pdf]

Supplementary Material

1 SUPPLEMENTARY DATA

Python source code for our analysis can be found on OSF. When using our scripts, please note that our methodology was developed and tuned on our clean in-lab data using the 80 s external auditory stimulus from the Explicit Accelerated Version of the task with two runs conducted. Functionalities, parameters, and thresholds may therefore need adjustments for other datasets and stimuli. It is particularly important that a representative speech envelope can be reconstructed from the participant recordings, for which however clean recordings are needed (consider pre-processing steps such as filtering).

2 SUPPLEMENTARY FIGURES AND TABLES

2.1 Figures

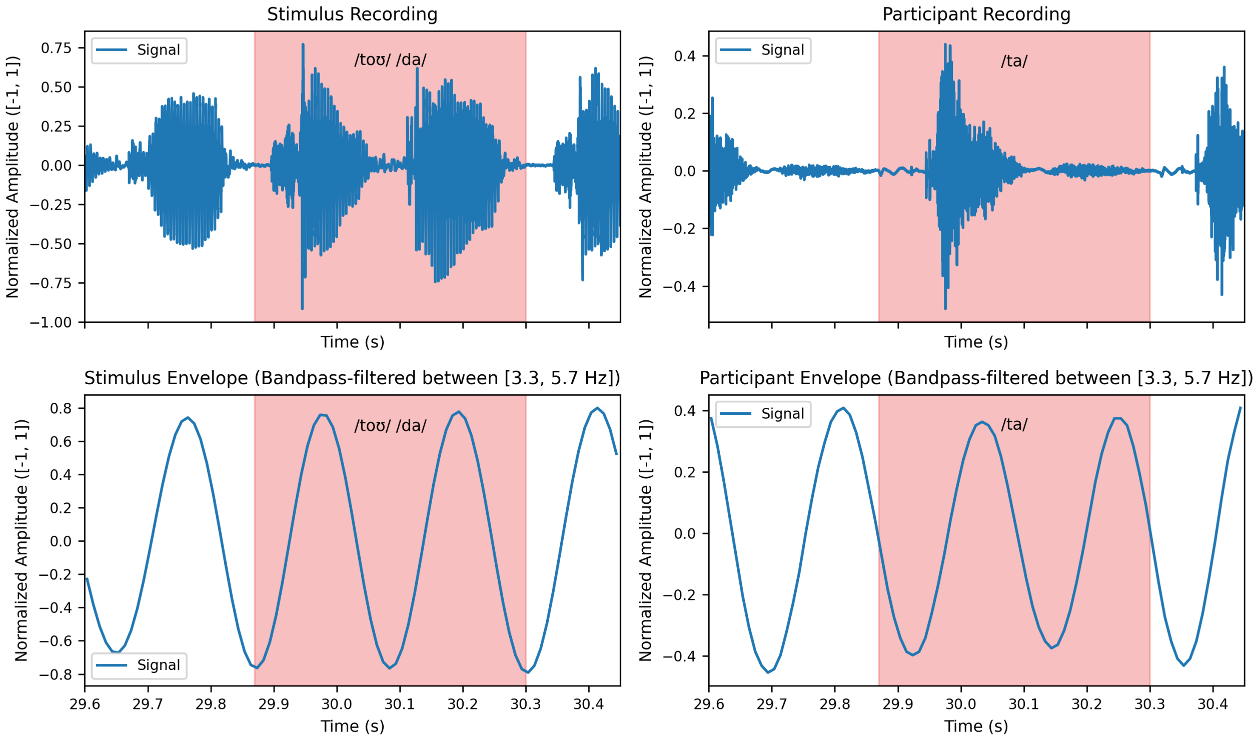

Figure S1. Filtered speech envelope of a subharmonic synchronizer (lower right) whispering /ta/ at half the syllable rate compared to the external auditory stimulus speech envelope (lower left). Both envelopes were filtered using a 5th order Butterworth bandpass filter (passband: 3.3-5.7 Hz). Comparing the envelopes to their respective speech waveforms (participant: upper right, external auditory stimulus: upper left), it appears that the /ta/ produced by the participant corresponds to actually two oscillations in the filtered speech envelope. This occurs due to the preservation of the second harmonic of the produced syllable rate and removal of the original syllable rate frequency because it falls outside the passband. For the external auditory stimulus, there is a one-to-one correspondence between the syllable in the waveform and its oscillation in the speech envelope, that is, one oscillation per syllable. When relying solely on speech envelopes, without reference to the speech waveform, it seems that the participant is matching the syllable rate of the auditory stimulus.

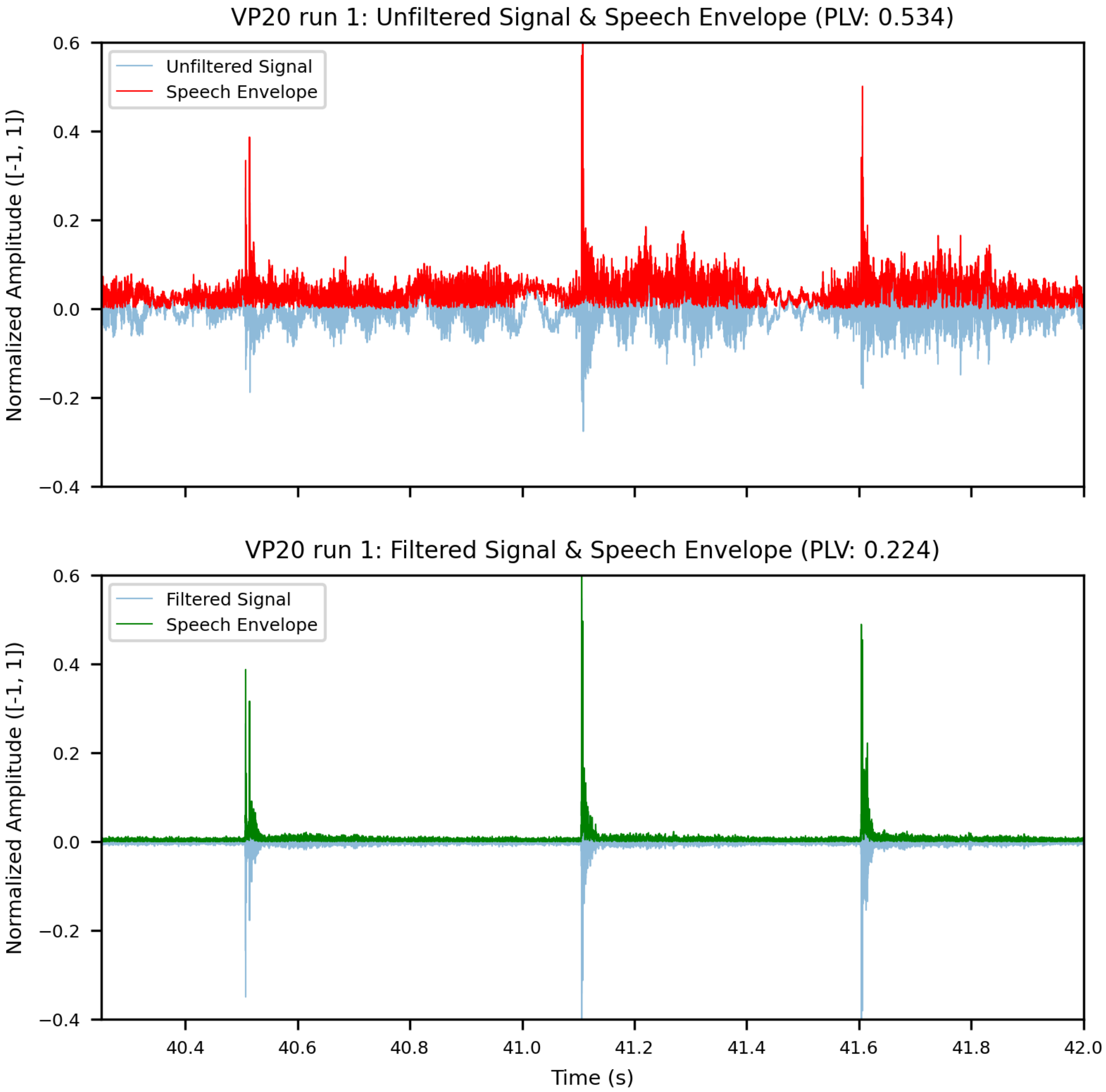

Figure S2. Exemplification of our script to compare pre- and post-filtered recordings and to validate the reconstruction of the speech envelope. Participant VP20 (from another independent sample using our scripts) was initially affected by a stimulus leakage and classified as a high-synchronizer with a PLV of 0.521. The upper plot displays the unfiltered recording, where the participant's actual classification as a subharmonic low-synchronizer (PLV post-filtered: 0.217, lower plot) is concealed by the leakage. The stimulus signal melted into the participant signal and left traces of its 200 Hz F0. Since the participant synchronized to only every second syllable in the stimulus, the stimulus leakage blended into the intervals between two consecutive participant syllables, resulting in estimated produced syllable rates that seemingly matched the stimulus syllable rates before filtering was applied. While the leakage is not clearly visible in the participant signal, it is prominently audible. This highlights that leakages that only blend into the signal—without visibly obscuring it—can yet heavily affect the signal's authenticity and analysis. After applying the bandstop filter, the lower plot shows improved distinction between the produced syllables and a clearer envelope which resulted in the correct estimation of participant's produced syllable rates (around 1.9 Hz). Note: The plots are zoomed in for enhanced clarity, although the script plots the entire recording and envelope.

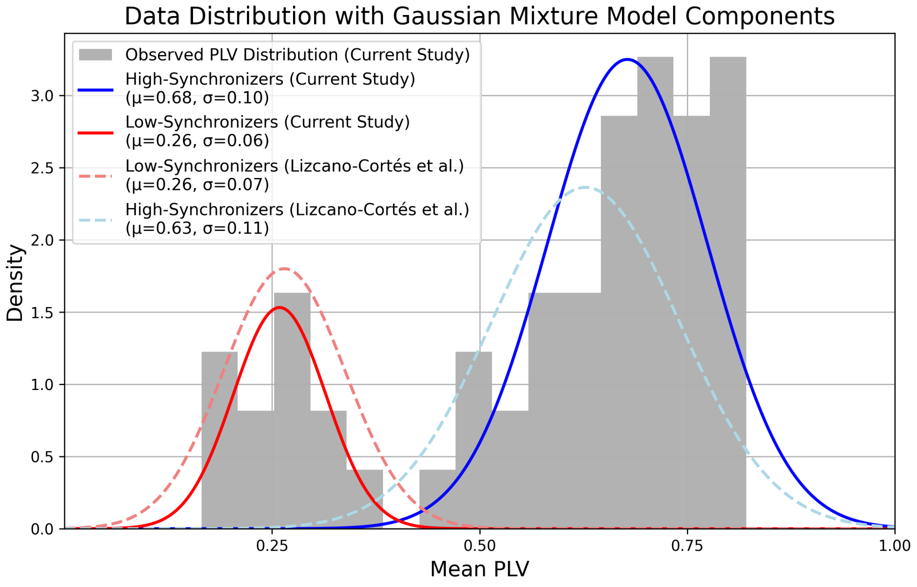

Figure S3. Density histogram of our sample (N = 56, N_{high} = 44, N_{low} = 12) showing the uneven distribution of high- and low-synchronizers after the removal of 4 participants. A two-component Gaussian Mixture Model (GMM) was fitted to our data and overlaid on the histogram, together with the GMM distribution reported by Lizcano-Cortés et al. (2022).

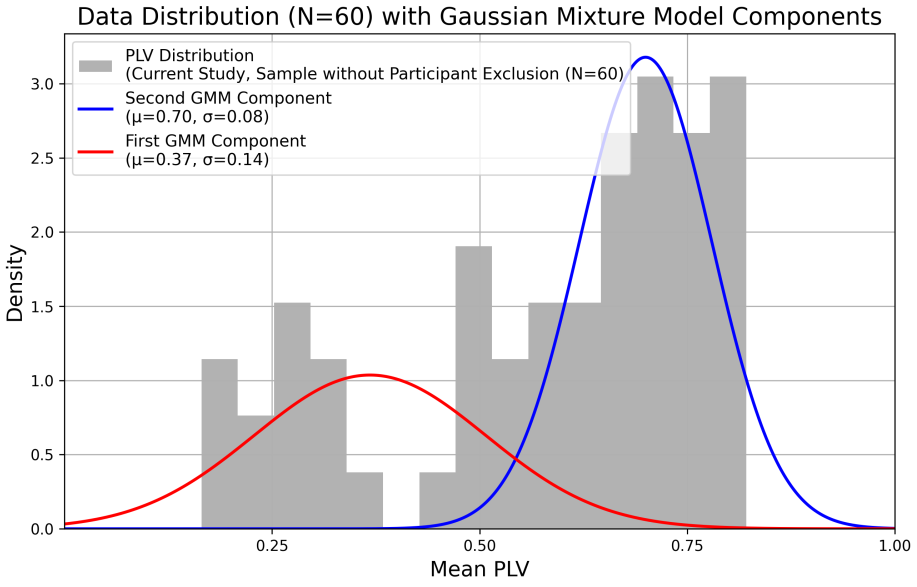

Figure S4. Density histogram of the full sample without exclusions (N = 60) with a two-component Gaussian Mixture Model (GMM) fitted to our data and overlaid on the histogram. Compared to Figure S3, the distribution of the first component is considerably broader, resulting in greater overlap and less distinct separation between the two components when all participants are included. Therefore, participants cannot be clearly classified as either high- or low-synchronizers. This further supports the validity of our exclusion criteria, as applying them improved the distinction between the two GMM components.

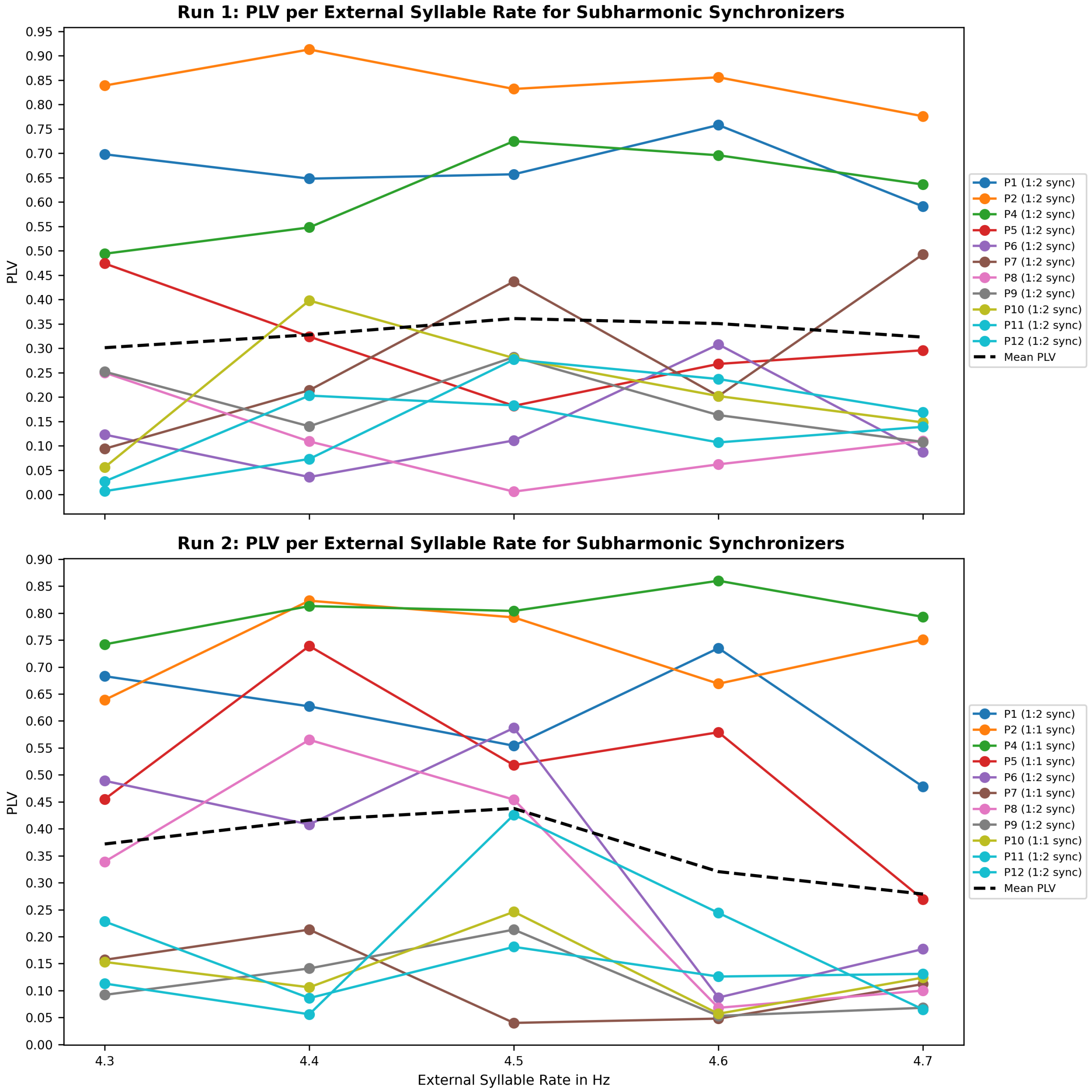

Figure S5. Segmented PLV (1:1 PLV computation, not the n:m PLV) for all individual subharmonic high- and low-synchronizers (cf. S1 and Figure 2 in main text). The dashed line represents the mean across all 11 participants. 1:2 sync = subharmonic synchronization, 1:1 sync = harmonic synchronization in the respective run. P5 and P6 are only included in this analysis for demonstrative purposes; they were discarded from the sample and all other analyses due to inconsistent classifications across runs.

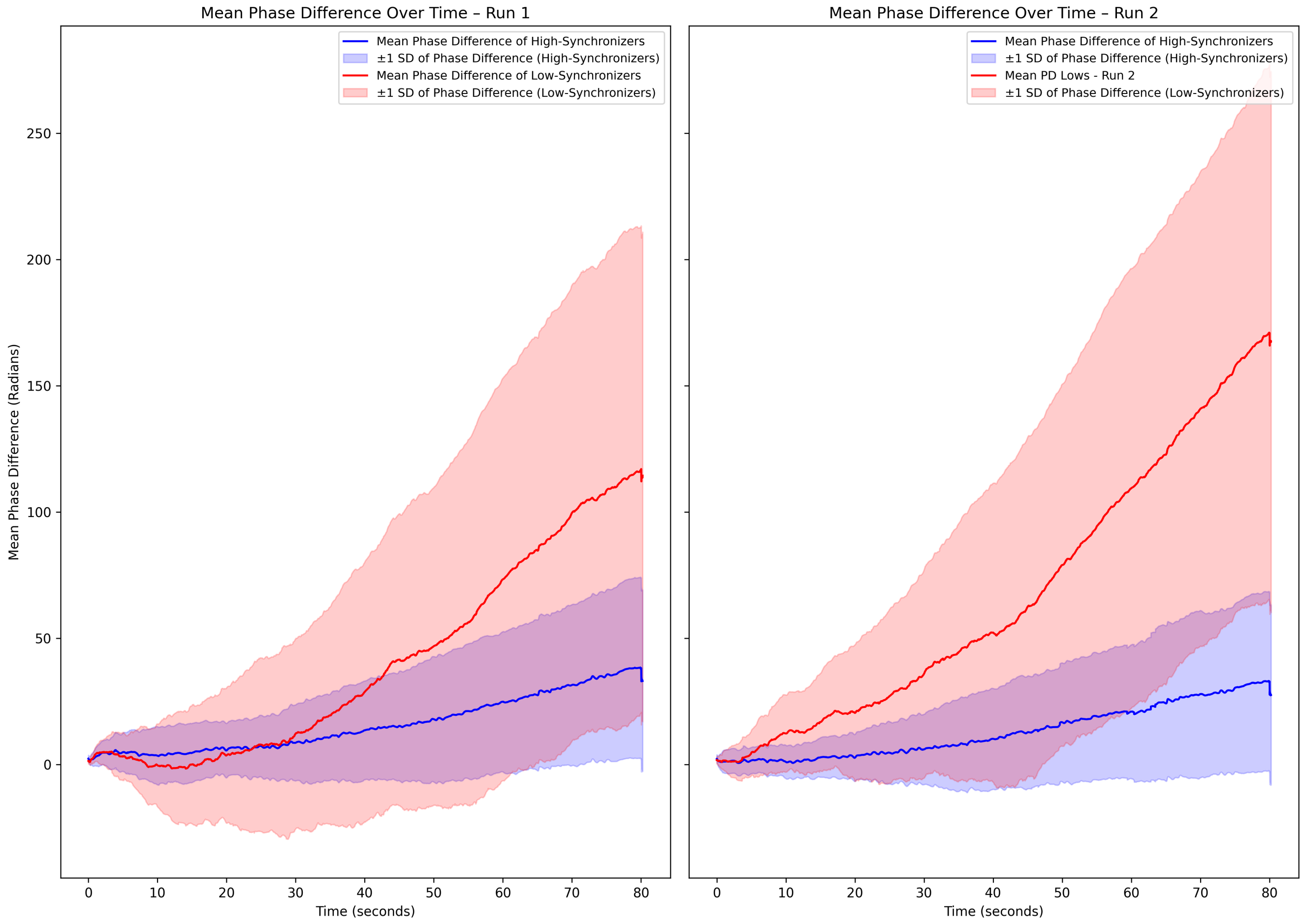

Figure S6. Mean phase difference over time between high- and low-synchronizers in response to the external auditory stimulus during run 1 (left) and 2 (right). High-synchronizers (blue) follow a more horizontal trend, that is, exhibit a more consistent phase difference over time. Low-synchronizers (red), on the other hand, show a steady accumulation of phase difference, particularly in run 2 and for the faster rates in the accelerating stimulus, resulting in their low PLVs.

2.2 Tables

Participant	PLV1	PLV2	Segment Syllable Rates Run 1	Segment Syllable Rates Run 2
P1	0.70	0.65	**[2.15, 2.20, 2.25, 2.30, 2.35]**	**[2.15, 2.20, 2.25, 2.30, 2.30]**
P2	0.86	0.76	**[2.15, 2.20, 2.25, 2.30, 2.35]**	[4.35, 4.40, 4.50, 4.55, 4.70]
P4	0.66	0.82	**[2.15, 2.20, 2.25, 2.30, 2.35]**	[4.30, 4.40, 4.50, 4.60, 4.70]
P5*	0.39	0.58	**[2.10, 2.10, 2.15, 2.10, 2.25]**	[4.25, 4.40, 4.50, 4.60, 4.60]
P6*	0.19	0.42	[1.80, 1.85, 1.90, 1.85, 1.85]	**[2.15, 2.10, 2.25, 2.10, 2.15]**
P7	0.41	0.22	**[2.70, 2.15, 2.20, 2.25, 2.35]**	[4.00, 3.85, 3.95, 3.90, 4.05]
P8	0.28	0.39	**[2.10, 2.05, 2.05, 1.85, 1.90]**	**[2.15, 2.20, 2.25, 2.25, 2.15]**
P9	0.33	0.21	**[1.50, 1.45, 1.60, 1.65, 1.55]**	**[2.00, 2.05, 2.10, 2.00, 1.95]**
P10	0.31	0.17	**[1.65, 2.25, 2.20, 2.05, 2.10]**	[3.15, 2.90, 2.95, 2.85, 2.90]
P11	0.23	0.27	**[2.50, 2.50, 2.45, 2.35, 2.35]**	**[1.95, 1.90, 2.10, 2.15, 2.05]**
P12	0.26	0.27	[**2.10, 1.75, 1.80, 1.65, 1.65]**	**[2.10, 1.90, 1.85, 1.85, 1.80]**

Table S1. PLVs and participant syllable rate frequencies of 11 subharmonic high- and low-synchronizers across runs. Perfectly subharmonic syllable rate frequencies per segment are given by [2.15, 2.20, 2.25, 2.30, 2.35]. While P1 almost perfectly 1:2 synchronized in run 1 and 2, P2 and P4 transition from almost perfect 1:2 synchronization to 1:1 synchronization in the second run. P2 achieves one of the highest PLVs observed in the data (0.86) via 1:2 synchronization. Participant runs synchronized close to or exactly at the subharmonic external syllable rate frequencies (highlighted in bold) and assigned PLVs according to which they would be classified as a high-synchronizer (cf. thresholds in the main text) can be regarded as "subharmonic high-synchronizers" (P1 run 1 and run 2, P2 run 1, P4 run 1), whereas others synchronizing at frequencies that deviate more significantly from the subharmonic rates and who were assigned low-synchronizer PLVs as "subharmonic low-synchronizers" (e.g., P11 in run 1, P10 in run 1). We further observed a 1:3 pattern in one subharmonic low-synchronizer (P9 in run 1 [articulation rate run 1: 1.55 Hz, stimulus articulation rate: ca. 4.55 Hz]; P9 run 2: 1:2 pattern). P7 and P11 in run 1 appear to start adapting more to subharmonic rates only after the first segment(s) (P7 starts too fast at 2.7 Hz, for instance). P5 shows moderate 1:2 synchronization in the first run (PLV: 0.39, classified as low-synchronizer) and stronger 1:1 synchronization in the second run (PLV: 0.58) and was thus able to be become a high-synchronizer in the second run—yet had to be excluded due to inconsistent classifications across runs, same as P6. PLVs were calculated following the procedure outlined in Section 2.4.3, based on the PLV formula presented in Section 2.1.4 in the main text that only accounts for 1:1 synchronization. Participants who 1:2 synchronize can be misleadingly assigned a high PLV due to the filtering issue described in 2.1.1 in the main text. For PLVs using the n:m PLV formula, see Table S2. Subharmonic synchronizers were identified as participants for whom, in at least one run, the syllable rate in any individual segment deviated no more than ±0.15 Hz from the corresponding predefined subharmonic reference rate ([2.15, 2.2, 2.25, 2.3, 2.35]). * = excluded participants

Participant	n:m PLV Run 1	n:m PLV Run 2
P1	0.74	0.65
P2	0.88	-
P4	0.62	-
P5*	0.39	-
P6*	0.19	0.38
P7	0.44	-
P8	0.27	0.36
P9	0.29	0.22
P10	0.31	-
P11	0.28	0.25
P12	0.20	0.21

Table S2. n:m PLVs (Vasudeva et al., 2022) accounting for 1:2 synchronization for the subharmonic synchronizers across both experiment runs. The expected subharmonic syllable rate frequencies lie between 2.15 and 2.35 Hz. Therefore, participant speech envelopes were zero-phase bandpass-filtered between 1.15 and 3.35 Hz (i.e., with a ±1 Hz margin) using a Butterworth filter of 5th order. In this analysis, all participant runs with produced non-subharmonic syllable rate frequencies (e.g., P2, P4, P5, and P7 in run 2, cf. Table S1) are excluded (-) since the 1:2 PLV cannot account for 1:1 synchronization, resulting in low PLVs even when 1:1 PLVs are high. The stimulus envelope was filtered as in the 1:1 synchronization analysis (i.e. a passband between [3.3, 5.7 Hz]), except that we additionally applied zero-phase filtering aiming to diminish phase distortions, given that the passbands for the participant and stimulus envelopes differ in the n:m synchronization analysis. For P9 run 1, the 1:3 PLV was used (and accordingly bandpass-filtered). PLVs for the subharmonically synchronized runs increased slightly using the n:m PLV compared to the 1:1 variant (e.g., P1, P2, P7 in run 1), remained the same (e.g., P1 run 2, P5 run 1), or decreased (e.g., P8 run 1, P12 run 2). * = excluded participants

3 EXTENDED DESCRIPTION AND DISCUSSION OF SUBHARMONIC SYNCHRONIZERS

Formerly, one additional participant run (P3, run2) was misclassified as a pure subharmonic synchronizer because the estimated syllable rates of that participant did not match the actually produced ones. This was due to them 1:1 synchronizing but emphasizing every second syllable in both runs, thus layering a 1:2 rhythm on top of the base rhythm that in the second run was actually more phase-aligning than the produced 1:1 rhythm in our PLV-based syllable rate estimation. Therefore, the syllable rate estimation confused the actual harmonic production in the second run with subharmonic rates.

We also investigated the modulation spectrum of this participant's speech envelope and found that the peak frequency occurred at the subharmonic (1:2 rhythm), as this is where the energy was strongest due to the consistent emphasis on every second syllable. Therefore, using the peak modulation frequency would have also led to a misclassification, yet even in both runs. Based on their synchronization behavior, this participant can be regarded as both a harmonic (1:1) and a subharmonic (1:2) synchronizer at the same time. What this behavior evidences reflects a mechanism known as hierarchical temporal processing in speech, that is, the simultaneous neural synchronization at multiple frequencies, a "key organizational aspect of the human cortical auditory system" (Peelle et al., 2010, p. 3). In auditory-motor synchronization, the speech motor cortex might also show simultaneous entrainment to multiple frequencies present in the input by the auditory cortex, which would explain the synchronization behavior of this participant.

Notably, P7, P11, and P4 (cf. Table S1) start with a non-subharmonic or more deviating rate compared to the stimulus. While P4 (run 1) begins producing syllables only 2.9 s after the stimulus started, they initially 1:1 synchronize for 9 syllables and then suddenly transition to 1:2 synchronization after a short breathing pause (this is not directly reflected in the estimated dominant syllable rate present in Table S1). P7 (run 1) starts rather early at 0.54 s but synchronizes at approximately 2.7 Hz in the first segment (stimulus: 4.3 Hz), then decelerates to a more subharmonically matching rate in the second segment (stimulus: 4.4 Hz, P7: approximately 2.15 Hz) and maintains the subharmonic pattern over the remaining segments. This suggests that P7, P11 and P4 exhibit initial entrainment challenges or flexibility in adapting to the stimulus, eventually stabilizing into a subharmonic synchronization pattern.

In subharmonic synchronizers, we further observed that they tend to produce more energetically stressed whispered syllables compared to the harmonic synchronizers, possibly due to more available time to articulate each syllable with emphasis. However, this emphasis might also help participants to better cope with the synchronization task by serving as a compensatory mechanism that enhances somatosensory feedback from oral articulators. Since participants are instructed to whisper and to turn up the volume of the headphones until they can no longer hear their own productions, the auditory feedback loop, which in normal speech is essential for self-monitoring and coordinating speech output, is diminished (cf. Bourhis et al., 2024, for the potential role of somatosensory feedback in reduced auditory feedback conditions). The reduced auditory feedback loop might therefore be a possible explanation for the emergence of subharmonic syllable synchronization, leading participants to produce slower, more stressed or emphasized rhythms to facilitate synchronization.

REFERENCES

Bourhis, M., Perrier, P., Savariaux, C., and Ito, T. (2024). Quick speech motor correction in the absence of auditory feedback. *Frontiers in Human Neuroscience* Volume 18 - 2024. doi:10.3389/fnhum.2024.1399316

Lizcano-Cortés, F., Gómez-Varela, I., Mares, C., Wallisch, P., Orpella, J., Poeppel, D., et al. (2022). Speech-to-speech synchronization protocol to classify human participants as high or low auditory-motor synchronizers. *STAR Protocols* 3, 1–9. doi:10.1016/j.xpro.2022.101248

Peelle, J. E., Johnsrude, I., and Davis, M. H. (2010). Hierarchical processing for speech in human auditory cortex and beyond. *Frontiers in Human Neuroscience* Volume 4 - 2010. doi:10.3389/fnhum.2010.00051

Vasudeva, B., Tian, R., Wu, D. H., James, S. A., Refai, H. H., Ding, L., et al. (2022). Multi-phase locking value: A generalized method for determining instantaneous multi-frequency phase coupling. *Biomedical Signal Processing and Control* 74, 103492. doi:10.1016/j.bspc.2022.103492
